# Character Strengths Across Disabilities: An International Exploratory Study and Implications for Positive Psychiatry and Psychology

**DOI:** 10.3389/fpsyt.2022.863977

**Published:** 2022-02-25

**Authors:** Emre Umucu, Beatrice Lee, Helen M. Genova, William J. Chopik, Connie Sung, Mizuka Yasuoka, Ryan M. Niemiec

**Affiliations:** ^1^Department of Counseling, Educational Psychology, and Special Education, Michigan State University, East Lansing, MI, United States; ^2^Center for Neuropsychology and Neuroscience Research, Kessler Foundation, West Orange, NJ, United States; ^3^Department of Psychology, Michigan State University, East Lansing, MI, United States; ^4^Education Director of the VIA Institute, VIA Institute on Character, Cincinnati, OH, United States

**Keywords:** disability, emotional disabilities, positive psychiatry, positive psychology, chronic condition

## Abstract

The purpose of this study was to examine the differences in character strengths for people with disabilities by using an international dataset by the VIA Institiute on Character. Specifically, we aimed to explore (a) the top and bottom five character strengths reported by different disability groups and all people with disabilities more broadly, and (b) group differences in each character strength and total character strengths. The investigator contacted the VIA Institute on Character (http://www.viacharacter.org/) for obtaining the dataset for the current study. After data cleaning, our sample size resulted in 11,699 people with disabilities. Among most people with disabilities, the top five character strengths scores were love of learning, honesty, appreciation of beauty and excellence, kindness, and fairness. The bottom five character strengths scores were self-regulation, perseverance, zest, spirituality, and prudence. Knowing that there is heterogeneity in character strengths across groups gives us a better understanding of the areas that people with different disabilities and conditions might thrive and provides clinicians and practitioners with a more nuanced understanding for how to possibly intervene with their clients. Positive psychiatry and psychology implications are discussed.

## Introduction

According to the World Health Organization (1), over one billion people worldwide are living with some form of disabilities and chronic conditions, which translates to ~15% of the world's population. The number of people with disabilities is increasing due to simultaneous increases in chronic health conditions and aging populations (1). People with disabilities can face different psychosocial challenges, such as functional limitations, secondary health conditions, social stigma, limited vocational functioning, and compromised community participation (2–8). Therefore, it is important to examine how to better support people with disabilities and chronic conditions and facilitate optimal psychosocial functioning through the lens of contemporary approaches such as positive psychiatry and psychology.

Shifting away from the traditional disease and pathology paradigm, positive psychology emphasizes building positive qualities rather than exclusively focusing on repairing weaknesses, aiming to understand what makes life worth living and enabling human thriving (9). Psychiatry has been defined as a subfield under medicine focusing on the diagnosis and treatment of mental illnesses (10). Recently, authors defined positive psychiatry as “the science and practice of psychiatry that seeks to understand and promote wellbeing through assessment and interventions aimed at enhancing behavioral and mental wellness” [(10), p. 2]. In recent decades, understanding disability and chronic conditions including mental illnesses has focused on holistic human functioning and the identification of human strengths and potentials (11).

Dr. Beatrice Wright, a pioneer in the rehabilitation field, promoted that psychosocial adjustment to disability and chronic conditions can be considered from a positive framework and postulated a coping vs. succumbing framework (12, 13). In this framework, *coping* focuses on an individual's positive qualities and abilities, whereas *succumbing* focuses on an individual's impairments and deficits (14). Consistent with the foundations of positive psychology, the rehabilitation field focuses on individuals with disabilities' capabilities and strengths (13, 15). Many studies have examined the effects of positive psychological characteristics (e.g., hope, resilience) on functioning and quality of life from a strength-based paradigm within the disability and rehabilitation field (5, 16–20). In the current study, we sought to illustrate variation in a particular classification of positive psychological characteristics—character strengths—in a large exploratory study of over 11,000 people with disabilities.

Character strengths are one of the foundations in the science of positive psychology (21, 22). Character strengths are defined as positive personality traits that reflect core identity, produce positive outcomes for oneself and others, and contribute to the collective good (21). Peterson and Seligman (23) developed *VIA Classification of Character Strengths* and classified 24 character strengths into six virtues: wisdom (i.e., creativity, curiosity, judgment, love of learning, perspective), courage (i.e., bravery, honesty, perseverance, zest), humanity (i.e., kindness, love, social intelligence), justice (i.e., fairness, leadership, teamwork), temperance (i.e., forgiveness, humility, prudence, self-regulation), and transcendence (i.e., appreciate of beauty and excellence, gratitude, hope, humor, spirituality). Character strengths are the psychological processes and mechanisms that depict virtues while virtues are described as the core moral characteristics that are universally valued (23). There is a large literature on the measurement, antecedents, and consequences of all 24 character strengths that suggest they play a critical role in positive psychological functioning and wellbeing [e.g., (23)]. Besides, Dahlsgaard et al. [(24), p. 2010] aimed to “create a consensual classification of human strengths while avoiding the criticism that any specific list we proposed would be culturally or historically idiosyncratic (23).” They reported that there is a convergence across place, time, and intellectual trandition about certain core virtues.

Growing research has examined the role of character strengths and character strength assessments in various disability groups, including individuals with intellectual and developmental disabilities (25, 26), multiple sclerosis [MS; (27)], traumatic brain injury [TBI; (28)], veterans with and without disabilities (29), and chronic conditions and disabilities (8). For instance, Niemiec et al. (25) discussed how building on character strengths can enhance support systems and quality of life of people with intellectual and developmental disabilities. Shogren et al. (26) examined the endorsement and psychometric properties of the *VIA Inventory of Strengths of Youth (VIA-Youth)* by exploring differences between youth with and without intellectual disability. Smedema (27) found that many character strengths played roles in the quality of life directly and indirectly through the negative effects of MS. Hanks et al. (28) found that character strengths and virtues were moderately associated with subjective wellbeing in people with mild to severe TBI. Umucu et al. (8) found that character strengths moderated the association between COVID-19 stress and the wellbeing among individuals with chronic conditions and disabilities.

More specifically, researchers have also been interested in examining the character strengths that were most frequently reported by people with disabilities. In a sample of adults with autism spectrum disorder, the most frequently reported character strengths were open-mindedness, creativity, and love of learning (30). Having a broader understanding of the character strengths reported by people with disabilities, including their commonalities and differences, provides us a broader insight into their psychological experiences, the sources of their wellbeing and quality of life, and provide a roadmap for moving forward in intervention work.

With the WHO's (1) emphasis on promoting rehabilitation services (e.g., psychiatric rehabilitation) and the increasing research attention on how character strengths make an impact on the lives of people with disabilities and chronic conditions, the purpose of this exploratory study was to examine the differences in character strengths for people with disabilities by using an international dataset by the VIA Institiute on Character. Specifically, we aimed to explore (a) the top and bottom five character strengths reported by different disability groups and all people with disabilities and chronic conditions more broadly, and (b) group differences in each character strength and total character strengths. To our knowledge, this is the first study using a large international sample to explore the differences in character strengths among people with different types of disabilities and chronic conditions including emotional conditions. Given positive psychiatry and psychology focuses on positive attributes and strengths, the results of the study will contribute to the existing international positive psychology, disability, and positive psychiatry literature on the role of character strengths in people with disabilities.

## Methods

### Procedure and Participants

This study was evaluated and approved by the Institutional Review Board of [blinded for review]. Upon approval of the study, the investigator contacted the VIA Institute on Character (http://www.viacharacter.org/) for obtaining the dataset for the current study. After data cleaning, our sample size resulted in 11,699 people with disabilities from across the globe.

### Measures

#### Sociodemographic Characteristics

Sociodemographic characteristics were measured via a demographic survey. Participants responded to questions regarding their age, gender, education, employment status, disability category, urban/rural living status, and countries. Regarding countries, although most participants reported their countries; some did not. More than half of the participants were from the US (>53.9%). There were participants from Canada (>6.7%), Australia (>6.0%), and United Kingdom (>4.9%), and Brazil (>3%). There were also participants from numerous other countries such as France, Estonia, Germany, Denmark, Belgium, Argentine, Turkey, India, Japan, Mexico, Netherlands, South Africa, and New Zealand.

#### Character Strengths

VIA Classification of Strengths and Virtues (character strengths) were measured using the VIA Inventory of Strengths—Positive [VIA-IS-P; (31)], which is a version of the original VIA-IS (23) but exclusively uses positively worded items. The VIA-IS-P consists of a total of 96 items, measuring 24 character strengths (4 items per character strengths). Each item is rated on a 5-point Likert-type scale ranging from 1(*Very Much Unlike Me*) to 5 (*Very Much Like Me*), with higher scores indicating higher character strengths. McGrath and Wallace (32) suggested researchers use the VIA-IS-P when there are concerns regarding participants' cognitive capacity to process negatively worded items. The mean alpha coefficient of the VIA-IS-P across strengths was 0.78 (32). The mean omega (ω) value of the VIA-IS-P was 0.78 (32). For our analyses, we examined disability group differences in each of the 24 character strengths and a composite measure in which all the character strengths were averaged together to yield one total score.

#### Disability Status

Disability status was asked with the following question: “Are you challenged by any of the following?” Participants had the option to select any disability status among multiple options (e.g., traumatic brain injury). We categorized disability types into eight categories, which included intellectual disabilities, sensory disabilities, emotional disturbance, orthopedic impairments, other health impairments, specific learning disabilities, traumatic brain injury, and multiple disabilities.

### Data Analysis

We conducted descriptive statistics to identify means and standard deviations for study variables. We used Shapiro–Wilk to check for data normality for each character strengths and total character strength scores. None of the scores fulfilled the criteria for normal distribution.

Next, we conducted Levene's test to examine homogeneity of variance on each dependent variable to test the assumption that individuals in each disability group varied in roughly homogeneous or similar ways. All of the Levene's tests were significant, showing that the groups did not have equal variances. As parametric assumptions were not met, we conducted a Kruskal–Wallis, non-parametric alternative to one-way ANOVA, to assess mean differences in character strengths and total character strengths among eight disability groups.

Finally, we used Dunn Bonferroni *post-hoc* tests to determine specific significant differences among groups. All analyses were conducted via SPSS 26.0 and DescTools (33), GmAMisc (34), and ggpubr (35) using the R Software (36).

## Results

### Descriptive Statistics

The majority of participants (~71%) were in the age range of 25–54. Most participants were women (72.3%), followed by men (25.7%) and others (2.0%). Regarding participants' education, about 72% of participants had at least a bachelor's degree. About 81% of participants were employed. Only 14.7% of participants reported they live in rural area. Regarding disability group, most participants had multiple disabilities (26.7%), followed by other health impairments (26.4%), emotional disturbance (23.1%), sensory disabilities (11.5%), specific learning disability (4.8%), orthopedic impairment (3.6%), intellectual disability (2.6%), and traumatic brain injury (1.2%). Table 1 demonstrates means and SDs for variables across disability groups.

**Table 1:** Demographic information and character strengths group characteristics and differences.

**Characteristics**	**Intellectual disabilities (IDG) *n* = 306**	**Sensory disabilities (SDG) *n* = 1,341**	**Emotional disturbances (EDG) *n* = 2,705**	**Orthopedic impairments (OIG) *n* = 426**	**Other health impairment *n* = 3,090**	**Specific learning disabilities (SLDG) *n* = 565**	**Traumatic brain injury (TBIG) *n* = 139**	**Multiple disabilities (MDG) n = 3,127**	**Test**
Women, *n* (%)	177 (57.8)	888 (66.2)	2,066 (76.4)	301 (70.7)	2,282 (73.9)	399 (70.6)	101 (72.7)	2,249 (71.9)	*p* < 0.05^a^
Virtues and Character Strengths									Group Differences Tests^b^
Wisdom, mean (SD)	19.57 (2.35)	19.31 (2.31)	18.89 (2.49)	19.43 (2.46)	19.38 (2.39)	19.49 (2.29)	19.89 (2.45)	19.40 (2.51)	*H^*a*^*= 96.46^*^
Creativity, mean (SD)	3.72 (0.87)	3.55 (0.79)	3.54 (0.82)	3.64 (0.77)	3.63 (0.82)	3.73 (0.81)	3.79 (0.79)	3.71 (0.80)	*H^*a*^*=93.60^*^
Curiosity, mean (SD)	3.93 (0.72)	3.90 (0.67)	3.77 (0.74)	3.91 (0.72)	3.89 (0.71)	4.00 (0.67)	4.01 (0.69)	3.89 (0.73)	*H^*a*^*= 82.74^*^
Judgment, mean (SD)	3.96 (0.62)	3.88 (0.58)	3.77 (0.64)	3.88 (0.63)	3.88 (0.59)	3.80 (0.59)	3.90 (0.58)	3.84 (0.63)	*H^*a*^*= 61.70^*^
Love of learning, mean (SD)	4.13 (0.66)	4.07 (0.65)	3.98 (0.72)	4.06 (0.67)	4.07 (0.68)	4.05 (0.65)	4.22 (0.65)	4.07 (0.69)	*H^*a*^*= 49.38^*^
Perspective, mean (SD)	3.81 (0.71)	3.88 (0.65)	3.81 (0.70)	3.93 (0.65)	3.89 (0.66)	3.88 (0.66)	3.95 (0.71)	3.87 (0.71)	*H^*a*^*= 28.88^*^
Courage, mean (SD)	13.30 (2.28)	13.78 (2.13)	12.80 (2.15)	2.17 (2.25)	13.37 (2.25)	13.53 (2.02)	14.44 (1.99)	13.17 (2.38)	*H^*a*^*= 282.81^*^
Bravery, mean (SD)	3.42 (0.84)	3.39 (0.77)	3.37 (0.81)	3.50 (0.75)	3.46 (0.81)	3.56 (0.75)	3.69 (0.80)	3.51 (0.83)	*H^*a*^*= 90.55^*^
Honesty, mean (SD)	3.98 (0.63)	4.09 (0.59)	3.93 (0.66)	4.09 (0.61)	4.03 (0.64)	3.96 (0.62)	4.25 (0.57)	4.00 (0.67)	*H^*a*^*= 91.94^*^
Perseverance, mean (SD)	2.89 (0.89)	3.09 (0.86)	2.76 (0.87)	3.10 (0.82)	2.85 (0.89)	2.73 (0.77)	3.20 (0.77)	2.77 (0.94)	*H^*a*^*= 211.03^*^
Zest, mean (SD)	3.00 (0.96)	3.21 (0.83)	2.73 (0.87)	3.18 (0.88)	3.01 (0.90)	3.28 (0.83)	3.29 (0.84)	2.87 (0.96)	*H^*a*^*= 408.61^*^
Humanity, mean (SD)	10.69 (2.18)	11.37 (1.71)	11.37 (1.76)	11.60 (1.73)	11.59 (1.73)	11.66 (1.65)	11.75 (1.72)	11.55 (1.82)	*H^*a*^*= 87.21^*^
Kindness, mean (SD)	3.91 (0.68)	3.98 (0.63)	3.97 (0.65)	4.03 (0.63)	4.02 (0.63)	4.06 (0.57)	4.03 (0.69)	4.08 (0.63)	*H^*a*^*= 54.96^*^
Love, mean (SD)	3.35 (1.12)	3.65 (0.92)	3.58 (0.96)	3.68 (0.92)	3.71 (0.93)	3.71 (0.92)	3.82 (0.84)	3.65 (0.97)	*H^*a*^*= 55.27^*^
Social intelligence, mean (SD)	3.43 (0.90)	3.73 (0.68)	3.81 (0.69)	3.88 (0.63)	3.85 (0.67)	3.89 (0.68)	3.89 (0.68)	3.81 (0.71)	*H^*a*^*= 98.37^*^
Justice, mean (SD)	10.53 (1.86)	11.08 (1.57)	10.66 (1.67)	11.17 (1.56)	11.02 (1.63)	11.19 (1.49)	11.46 (1.76)	10.85 (1.75)	*H^*a*^*=144.57^*^
Fairness, mean (SD)	3.98 (0.69)	4.00 (0.66)	3.89 (0.74)	4.00 (0.63)	4.00 (0.69)	4.02 (0.67)	4.12 (0.67)	3.98 (0.71)	*H^*a*^*= 45.77^*^
Leadership, mean (SD)	3.15 (1.03)	3.46 (0.87)	3.31 (0.92)	3.61 (0.88)	3.47 (0.89)	3.56 (0.82)	3.65 (0.96)	3.38 (0.93)	*H^*a*^*=118.40^*^
Teamwork, mean (SD)	3.39 (0.81)	3.61 (0.69)	3.46 (0.73)	3.54 (0.65)	3.54 (0.72)	3.60 (0.67)	3.69 (0.66)	3.48 (0.76)	*H^*a*^*= 72.83^*^
Temperance, mean (SD)	13.36 (2.15)	13.81 (2.02)	13.07 (2.15)	13.75 (2.05)	13.43 (2.14)	13.05 (2.05)	13.86 (2.05)	13.19 (2.28)	*H^*a*^*= 156.20^*^
Forgiveness, mean (SD)	3.58 (0.79)	3.67 (0.73)	3.54 (0.77)	3.72 (0.68)	3.68 (0.75)	3.71 (0.70)	3.65 (0.69)	3.63 (0.78)	*H^*a*^*= 64.65^*^
Humility, mean (SD)	3.47 (0.72)	3.59 (0.67)	3.51 (0.73)	3.49 (0.65)	3.54 (0.71)	3.44 (0.71)	3.60 (0.67)	3.51 (0.72)	*H^*a*^*= 26.44^*^
Prudence, mean (SD)	3.43 (0.85)	3.55 (0.79)	3.37 (0.88)	3.51 (0.83)	3.43 (0.83)	3.24 (0.82)	3.52 (0.81)	3.36 (0.88)	*H^*a*^*= 85.74^*^
Self-regulation, mean (SD)	2.87 (0.87)	2.99 (0.84)	2.63 (0.89)	3.02 (0.84)	2.76 (0.90)	2.64 (0.84)	3.08 (0.87)	2.68 (0.93)	*H^*a*^*= 232.17^*^
Transcendence, mean (SD)	17.62 (3.06)	18.42 (2.72)	17.18 (2.90)	18.68 (2.92)	18.14 (2.97)	18.16 (2.71)	17.91 (3.10)	17.91 (3.10)	*H^*a*^*= 273.37^*^
Appreciation of beauty and excellence, mean (SD)	3.93 (0.84)	3.99 (0.70)	4.01 (0.73)	4.06 (0.70)	4.02 (0.73)	4.00 (0.72)	4.14 (0.69)	4.05 (0.74)	*H^*a*^*= 21.20^*^
Gratitude, mean (SD)	3.34 (0.86)	3.64 (0.78)	3.26 (0.83)	3.64 (0.83)	3.53 (0.84)	3.53 (0.78)	3.80 (0.78)	3.42 (0.88)	*H^*a*^*= 284.89^*^
Hope, mean (SD)	3.49 (0.83)	3.68 (0.72)	3.19 (0.84)	3.71 (0.75)	3.54 (0.81)	3.60 (0.74)	3.78 (0.70)	3.44 (0.87)	*H^*a*^*= 501.52^*^
Humor, mean (SD)	3.71 (0.90)	3.73 (0.86)	3.57 (0.92)	3.84 (0.92)	3.74 (0.86)	3.78 (0.85)	3.75 (0.87)	3.72 (0.89)	*H^*a*^*= 82.37^*^
Spirituality, mean (SD)	3.13 (1.11)	3.35 (1.01)	3.14 (1.01)	3.41 (1.02)	3.28 (1.04)	3.23 (1.01)	3.55 (0.97)	3.33 (1.03)	*H^*a*^*= 87.84^*^
Character strengths, mean (SD)	85.10 (9.84)	87.80 (9.24)	84.00 (9.40)	88.55 (9.86)	86.95 (9.80)	87.11 (8.69)	90.48 (9.22)	86.08 (10.57)	*H^*a*^*= 255.79^*^

a *Chi-square test*.

b *Kruskal–Wallis H test*.

* *p < 0.05*.

### Character Strengths Profiles

**Figure 1:**
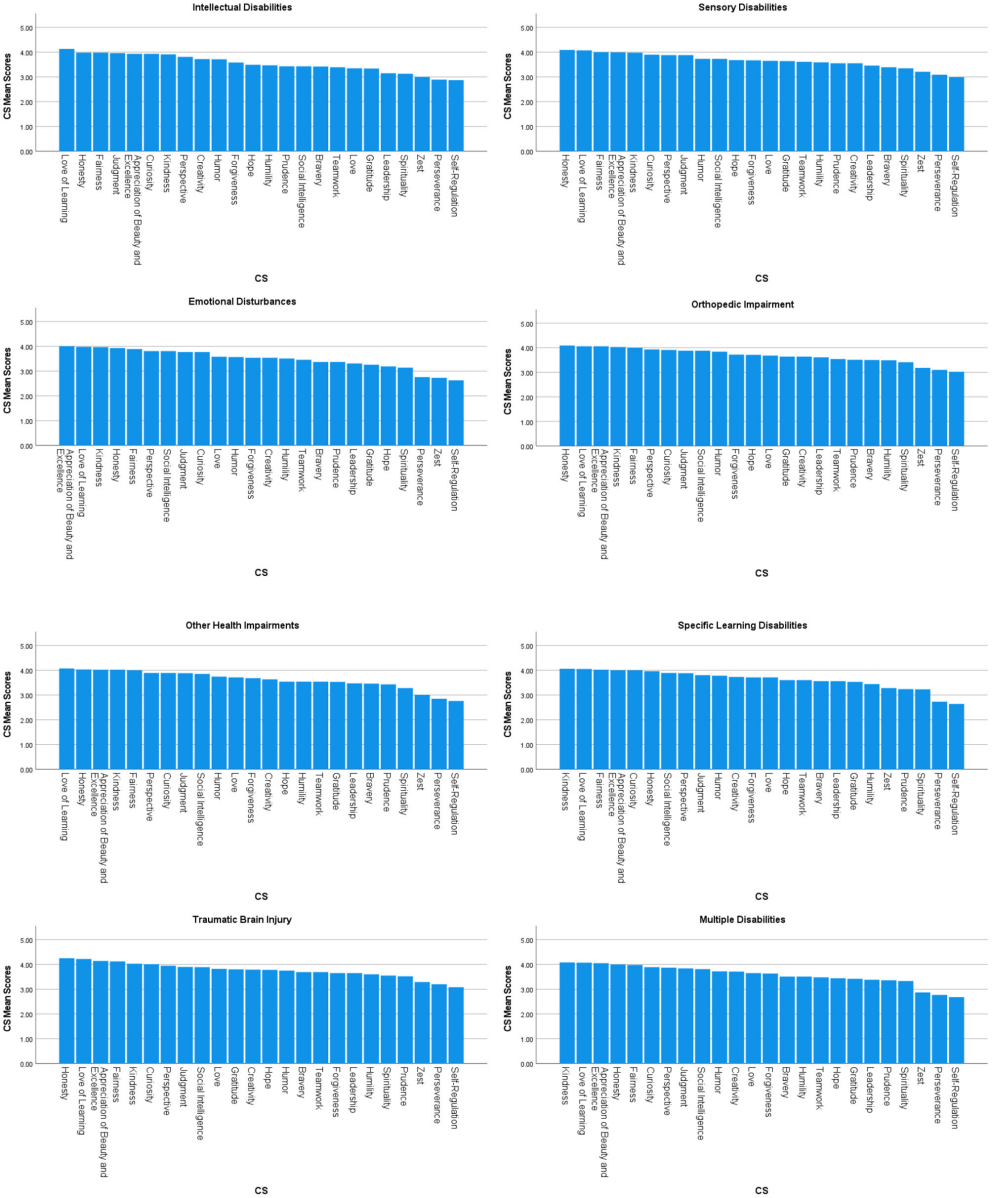
Character strengths profiles for each disability group.

**Figure 2:**
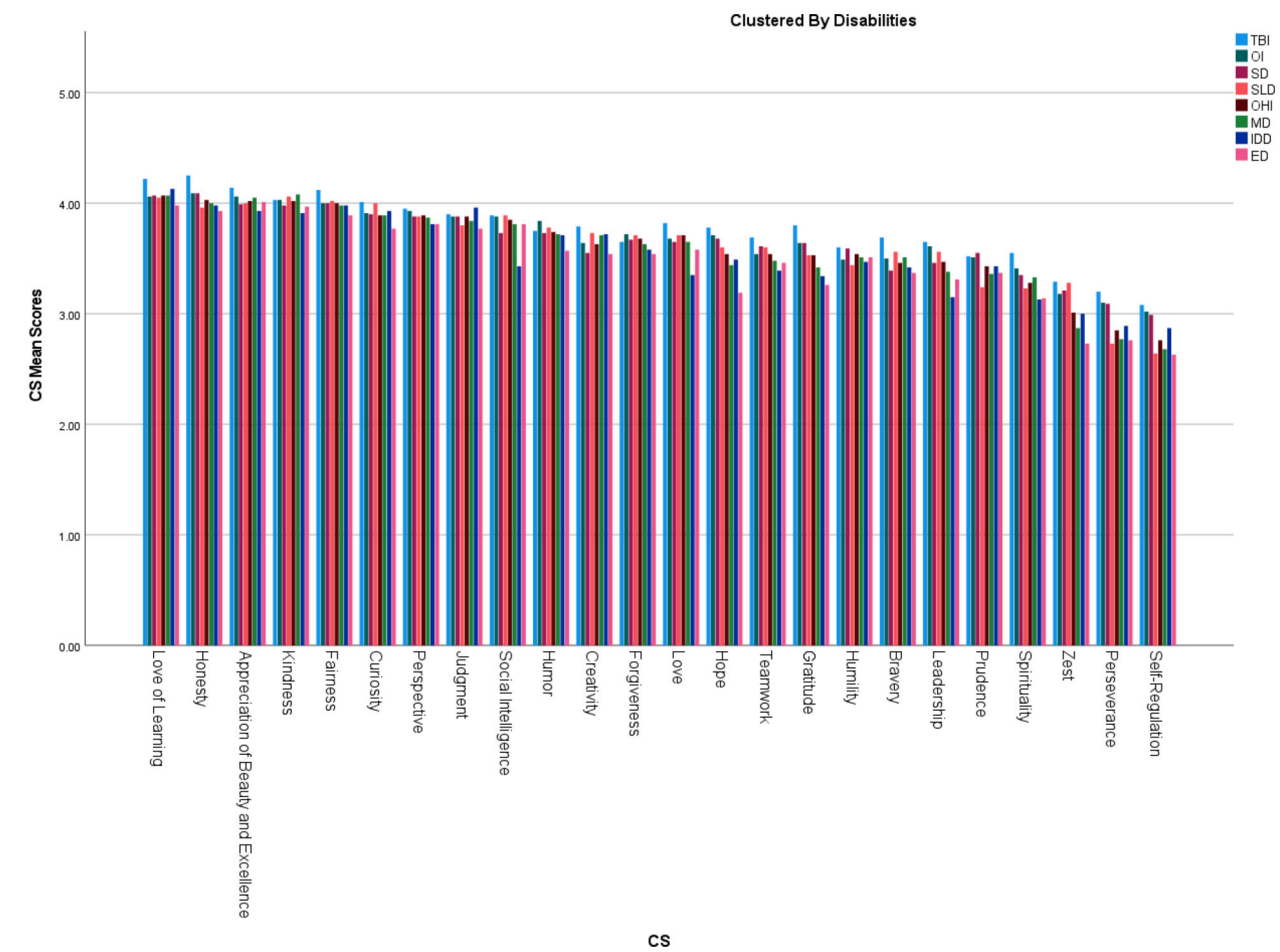
Overall character strengths profile. TBI, Traumatic brain injury; OI, Orthopedic impairment; SD, Sensory disabilities; SLD, Speech-language disabilities; OHI, Other health impairments; MD, multiple disabilities; IDD, Intellectual disabilities; ED, Emotional disturbance.

**Figure 3:**
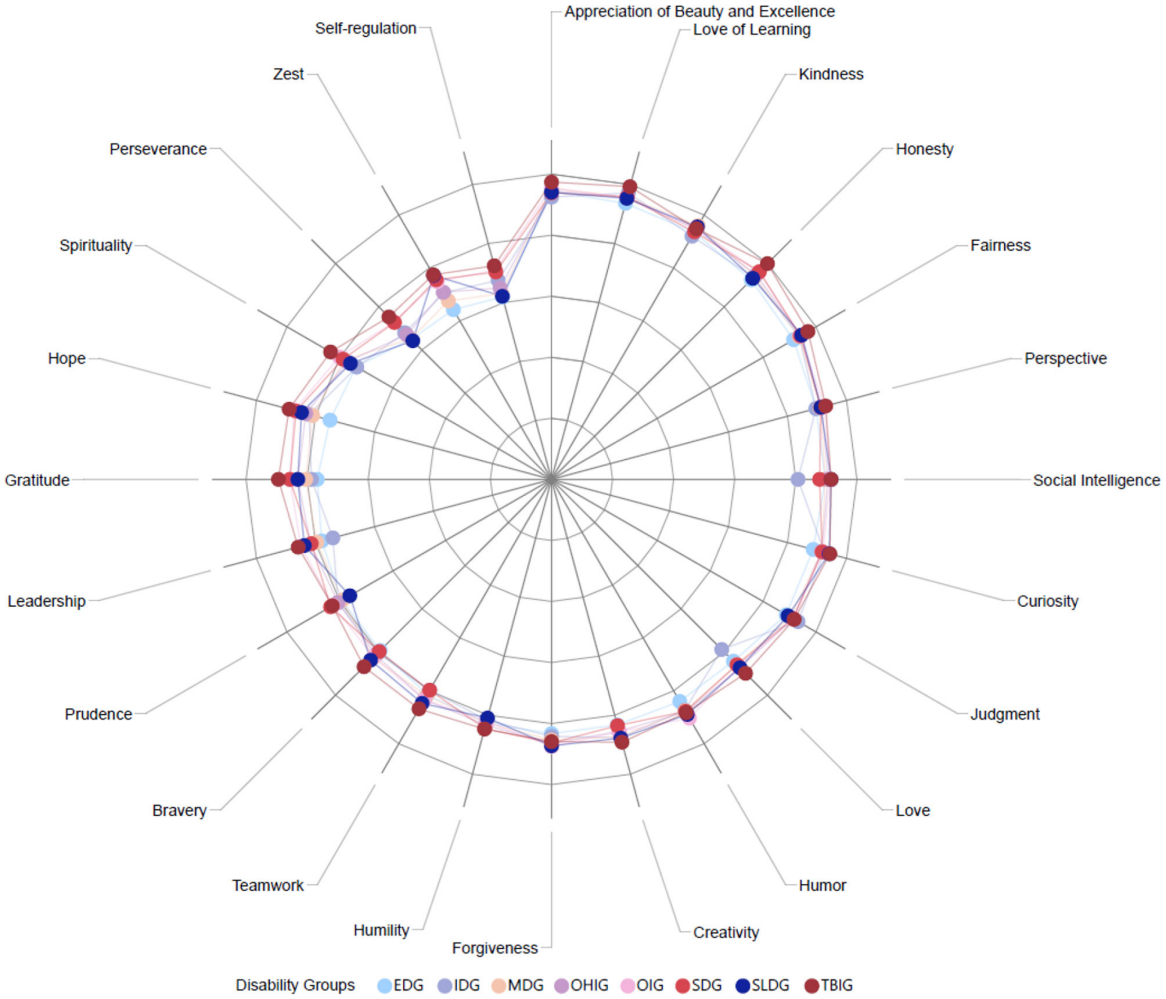
Radar chart for disability groups by character strengths. TBI, Traumatic brain injury; OI, Orthopedic impairment; SD, Sensory disabilities; SLD, Speech-language disabilities; OHI, Other health impairments; MD, multiple disabilities; IDD, Intellectual disabilities; ED, Emotional disturbance.

We created character strengths profiles for each disability based on participants' mean scores for each CS scores. Figures 1, 2 demonstrate character strengths profiles for each disability group and overall character strengths profile. Figure 3 represents radar chart for disability groups by character strengths.

#### Intellectual Disability Group (IDG; 2.6%)

Among people with intellectual disabilities, the top five character strengths scores were love of learning, honesty, fairness, judgment, and appreciation of beauty and excellence. The bottom five character strengths scores were self-regulation, perseverance, zest, spirituality, and leadership.

#### Sensory Disability Group (SDG; 11.5%)

Among individuals with sensory disabilities, the top five character strengths scores were honesty, love of learning, fairness, appreciation of beauty and excellence, and kindness. The bottom five character strengths scores were self-regulation, perseverance, zest, spirituality, and bravery.

#### Emotional Disturbances Group (EDG; 23.1%)

Among people with emotional disturbances, the top five character strengths scores were appreciation of beauty and excellence, love of learning, kindness, honesty, and fairness. The bottom five character strengths scores were self-regulation, zest, perseverance, spirituality, and hope.

#### Orthopedic Impairment Group (OIG; 3.6%)

Among people with orthopedic disabilities, the top five character strengths scores were honesty, love of learning, appreciation of beauty and excellence, kindness, and fairness. The bottom five character strengths scores were self-regulation, perseverance, zest, spirituality, and humility.

#### Other Health Impairments Group (OHIG; 26.4%)

Among people with other health impairments, the top five character strengths scores were love of learning, honesty, appreciation of beauty and excellence, kindness, and fairness. The bottom five character strengths scores were self-regulation, perseverance, zest, spirituality, and prudence.

#### Specific Learning Disabilities Group (SLDG; 4.8%)

Among people with specific learning disabilities, the top five character strengths scores were love of learning, honesty, fairness, judgment, and appreciation of beauty and excellence. The bottom five character strengths scores were self-regulation, perseverance, zest, spirituality, and leadership.

#### Traumatic Brain Injury Group (TBIG; 1.2%)

Among people with TBI, the top five character strengths scores were honesty, love of learning, appreciation of beauty and excellence, fairness, and kindness. The bottom five character strengths scores were self-regulation, perseverance, zest, prudence, and spirituality.

#### Multiple Disability Group (MDG; 26.7%)

Among people with multiple disabilities, the top five character strengths scores were kindness, love of learning, appreciation of beauty and excellence, honesty, and fairness. The bottom five character strengths scores were self-regulation, perseverance, zest, spirituality, and prudence.

#### Summary

Among all people with disabilities, the top five character strengths scores were love of learning, honesty, appreciation of beauty and excellence, kindness, and fairness. The bottom five character strengths scores were self-regulation, perseverance, zest, spirituality, and prudence.

### Group Differences in Character Strengths Score

Kruskal–Wallis test results revealed that there were statistically significant differences in total character strengths score among the eight disability groups, χ^2^(7) = 255.78, *p* < 0.05. Please see Appendix for group differences in each character strength score.

### Dunn Bonferroni *post-hoc* Analyses

Follow-up Dunn's *pos-hoc* tests were conducted to examine pairwise differences among the disability groups. Significance values have been adjusted by the Bonferroni correction for multiple tests. Regarding total character strengths (CS) total scores, SDG [mean rank difference (MRD) = 924.44, *p* < 0.05], OIG (MRD = 1255.23, *p* < 0.05), OHIG (MRD = 662.52, *p* < 0.05), and TBIG (MRD = 1864.94, *p* < 0.05) had significantly higher CS total scores compared with IDG. SDG (MRD = 1298.14, *p* < 0.05), OIG (MRD = 1628.94, *p* < 0.05), OHIG (MRD = 1036.23, *p* < 0.05), SLDG (MRD = 1073.41, *p* < 0.05), TBIG (MRD = 2238.65, *p* < 0.05), and MDG (MRD = 722.90, *p* < 0.05) had significantly higher CS total scores compared with EDG. OHIG (MRD = −592.71, *p* < 0.05) had significantly lower CS total score compared with OIG. TBIG had significantly higher CS total scores compared with OHIG (MRD = 1202.41, *p* < 0.05), SDG (MRD = 940.50, *p* < 0.05), and SLDG (MRD = 1165.23, *p* < 0.05). MDG had significantly lower CS total scores than SDG (MRD = −575.24, *p* < 0.05), OIG (MRD = −906.03, *p* < 0.05), OHIG (MRD = −313.32, *p* < 0.05), and TBIG (MRD = −1515.74, *p* < 0.05). Please see Appendix and Table 1 for Dunn Bonferroni *post-hoc* Analyses results for each CS.

## Discussion

This study is the first attempt, to our knowledge, to examine character strengths in individuals across a wide range of disability and chronic condition groups around the world. Growing research has suggested that positive approaches to psychological treatment (such as increasing awareness and usage of character strengths) in individuals with disabilities may be a powerful asset in meeting therapeutic goals and increasing quality of life and wellbeing (8, 11, 25, 29). An alternative to deficit-based methods, which attempt to reduce negative behaviors or symptoms associated with a given disability, character strengths provide a language with which individuals can focus on the aspects of themselves that potentially benefit themselves and society (25). The current study used a well-validated and widely-used classification system and its measurement tool, the *VIA Inventory of Strengths*, in a large international sample across multiple disability types, including emotional conditions and disabilities.

One of the strengths of the study was the inclusion of individuals with physical, cognitive, and emotional disabilities. In addition, disabilities occurring at different developmental stages of life are represented in the sample. For example, intellectual disabilities, like autism, constitute disabilities that emerge relatively early in life and have an enduring presence. The character strengths of individuals with these disabilities might differ compared to individuals with more acute or sudden/adult-onset disabilities, like a traumatic brain injury. Interestingly, we found that commonly reported character strengths in individuals with disabilities include love of learning, honesty, appreciation of beauty and excellence, kindness, and fairness. We found that the least commonly reported strengths in our disability sample included self-regulation, perseverance, zest, spirituality, and prudence. A previous study (27) found the top five strengths were honesty, kindness, fairness, humor, and gratitude, and the bottom five strengths were forgiveness, humility, spirituality, zest, and self-regulation in people with multiple sclerosis. In another study, authors found that the most common character strengths were curiosity, fairness, kindness, judgment, honesty, and leadership, while the least common character strengths were zest, prudence, perseverance, humility, hope, self-regulation, and spirituality in people with dysclexia (37). These results are partially consistent with our findings; however, it is also important to highlight that the most and least common character strengths may change based on disability groups.

Generally, individuals within the TBI group rated their strengths higher overall than other disability groups. For example, the TBI group's endorsements of creativity, curiosity, love of learning, bravery, honesty, perseverance, zest, love, social intelligence, fairness, leadership, prudence, self-regulation, gratitude, hope, and spirituality were significantly higher compared to other disability groups. The only character strength in which the TBI group was lower than others was teamwork, in which they were significantly lower than the MDG group, but significantly higher than IDG group. No significant differences were observed between TBI and other disability groups in judgement, perspective, kindness, forgiveness, humility, appreciation of beauty, excellence, and humor. These findings may provide some interesting avenues of future research in the TBI field. Given that individuals with TBI can often experience mood issues (38, 39), reduced quality of life (40–42), and reduced wellbeing (43), using character strengths knowledge and awareness appears to be an interesting treatment avenue to address these concerns.

One important consideration is that individuals with TBI can have significant self-awareness issues, including both under- and overestimating their skills and abilities (44–46). Therefore, endorsing strengths higher than other comparison groups may be indicative of this phenomenon. However, it would not be useful to dismiss these endorsements as “self-awareness” discrepancies in the TBI group. Rather, in the future, it may be useful to also assess the strengths of an individual with TBI through the perceptions of a trusted “other” who may give a realistic profile of an individual's strengths (e.g., friends, family). Having both the perception of the client as well as a significant other may elucidate which strengths the person expresses in daily life, which may aid in development of therapeutic goals, and which strengths might be inflated because of their limitations in accurately evaluating their strengths.

Those with emotional disturbances reported lower character strengths than other disability groups, which may reflect the poorer self-esteem and lower self-concept commonly observed in those with depression and anxiety. This lower endorsement of character strengths, however, may also provide an opportunity for clinicians to utilize strength identification in their therapy goals. Those with emotional disturbance may be less aware of strengths and therefore merely the identification of strengths may be a powerful exercise.

Another group with low endorsement of character strengths was the IDG group. There is a growing consensus in the field of intellectual disabilities, including autism, that using only a deficit-based approach when treating individuals with autism can compromise self-esteem and leave individuals unaware of their strengths. Therefore, recently a shift has emerged in the autism field to focus on not only strength-based approaches but character strengths-based approaches (25, 30, 47). Our findings indicated lower strength ratings in this group compared to other disability groups indicates that indeed, strength identification needs bolstering in this group. Interestingly, their love of learning endorsements was higher than other disability groups, which may reflect a commonly reported trait of autism: restricted interests. Specifically, those with autism may seek information about a restricted topic to a greater degree than what is typically observed in others, which is generally referred to as a “symptom” or “challenge.” However, reframing this trait as a love of learning may be beneficial as it could help autistic individuals, as well as others, understand how this trait may benefit society as well.

Some strength endorsements may reflect the commonly observed traits in individuals with disabilities. For example, low levels of zest, which was consistently one of the lowest reported strengths of the current study may be correlated with significant fatigue levels observed in individuals with disabilities. Fatigue is one of the most commonly reported symptoms in clinical care in the general population and is one of the most commonly reported symptoms in individuals with illness or disability. Thus, low levels of zest may be related to the significant feelings of fatigue felt by this population. In addition, previous literature reported that zest was one of the lowest five character strengths among university students (48) and people with disabilities (27). While increasing zest may represent a treatment target for positive psychology interventions, it may also be helpful for clinicians to keep in mind that chronic fatigue due to illness, disability, comorbidity, medications, and other reasons may make it difficult for certain disability groups to unilaterally increase their zest and energy levels. Rather, setting goals and working on the strengths of perseverance or self-regulation may be more useful, as they involve making choices to overcome obstacles or making choices (such as conserving energy). Together, these proximal goals may be more realistic for someone suffering from fatigue.

Our study findings may provide some significant clinical implications for the use of character strengths interventions in the fields of rehabilitation, psychiatry, and psychology for people with disabilities and chronic conditions. Previous research has demonstrated positive associations between character strengths and subjective health status (49). In a cross-cultural longitudinal study, researchers found that using character strengths is not only beneficial for self-perceived physical health when going through difficult situations, but using strengths is also for enhancing meaning in life, social connectedness, and mental health (50). Given the association between character strengths and health outcomes, clinicians may include interventions such as *using signatures in a new way* or *identifying signature strengths* (51) and *strengths reframing* and the *aware-explore-apply model of strengths* (25) when working with people with disabilities. By helping people with disabilities and chronic conditions identify and utilize their personal character strengths, this may potentially improve their perceived health, psychosocial functioning, and quality of life.

In addition, given this is an international study with multiple countries and disabilities [we consider each disability category as a cultural subgroup (e.g., deaf culture)], it is important to consider cross-cultural and demographic differences in character strengths. If each disability subgroup is considered a cultural subgorup, we suggest each disability group may, on average, have different top and bottom character strengths. For example, people with physical disabilities may experience or express character strengths different compared to people with psychiatric disabilities, just as individuals from different countries and cultures may express character strengths in a different ways reflecting their culture.

Positive psychiatry and psychology aims to examine positive attributes and strengths (10). Besides, positive psychiatry researchers focuses on protective psychosocial factors in chronic conditions. Given our research (a) identifies and describes character strengths in people with chronic conditions, including emotional conditions and (b) demonstrates the top and bottom character strengths among different disability subgroups, findings from this study may contribute to clinical and research practice. This research will help health professionals understand what character strengths are being used among people with different disabilities, which could be helpful in developing tailored and personalized interventions.

There are several limitations that should be considered in this study. The study comprised a convenience sample of individuals with self-reported disabilities, and some participants may not have accurately reported their disability status. As functional and cognitive abilities were not directly assessed, participants' responses may not accurately reflect their perceived character strengths and virtues when navigating an online survey. Further, the ability to complete the survey (and even have access to the survey) assumes that we may have recruited a higher functioning sample; however, there was no way to evaluate this concern. We interpreted the findings by considering that people with learning disabilities, intellectual disabilities, and TBI may have experienced difficulties understanding some concepts and questions. The majority of participants were women, had at least a bachelor's degree, and were employed, which could also limit the generalizability of this study's findings to the general disability population. Another limitation in this study was that participants were categorized into eight disability groups. A more systematic way of categorizing various disabilities could be beneficial. Also, some specific disability types may have been left out and not enough participants were present in different countries for us to formally model between-country variation in the disability group differences that we observed. Future studies are warranted to further examine character strengths in people with disabilities around the globe. We interpreted the findings considering the demographic characteristics of the respondents. For example, the majority of participants were in the age range of 25–54. Therefore, we cannot generalize our findings for all age groups. Similarly, the majority of participants were women, which decreases our ability to generalize our findings. Gender differences are especially important given previous research reported that women typically score higher on strengths than men although the top five strengths were similar among men and women (52).

In conclusion, it is important to highlight that we comment on what has been established in this unique data set and also summarize future considerations to be explored although we are aware that there are many important points to be highlighted in this study. Character strengths has been recently examined in different disability populations (3, 8, 25, 53, 54). In addition to other studies, our study provided a greater understanding of character strengths in a heterogeneous international sample of individuals with disabilities. Overall, this sample of people with disabilities reported their top five character strengths as love of learning, honesty, appreciation of beauty and excellence, kindness, and fairness, and reported their bottom five character strengths as self-regulation, perseverance, zest, spirituality, and prudence. Knowing that there is heterogeneity in character strengths across groups gives us a better understanding of the areas that people with different disabilities might thrive and provides clinicians and practitioners with a more nuanced understanding for how to possibly intervene with their clients.

## Data Availability Statement

The original contributions presented in the study are included in the article/supplementary material, further inquiries can be directed to the corresponding author/s.

## Author Contributions

All authors listed have made a substantial, direct, and intellectual contribution to the work and approved it for publication.

## Conflict of Interest

The authors declare that the research was conducted in the absence of any commercial or financial relationships that could be construed as a potential conflict of interest.

## Publisher's Note

All claims expressed in this article are solely those of the authors and do not necessarily represent those of their affiliated organizations, or those of the publisher, the editors and the reviewers. Any product that may be evaluated in this article, or claim that may be made by its manufacturer, is not guaranteed or endorsed by the publisher.
