# Supplementary material for: Character Strengths Across Disabilities: An International Exploratory Study and Implications for Positive Psychiatry and Psychology
Source: Front Psychiatry. 2022 Feb 25;13:863977. doi: 10.3389/fpsyt.2022.863977 (PMC8914428; doi:10.3389/fpsyt.2022.863977)
Supplement: Supplementary file 1 [file Data_Sheet_1.docx]

**Group Differences in Each Character Strength Score**

Kruskal-Wallis test results revealed that there were statistically significant differences in each character strength score among the 8 disability groups, *χ*^2^(7) = 90.55, *p* < 0.05 for *bravery*; *χ*^2^(7) = 55.27, *p* < 0.05 for *love*; *χ*^2^(7) = 85.74, *p* < 0.05 for *prudence*; *χ*^2^(7) = 72.83, *p* < 0.05 for *teamwork*; *χ*^2^(7) = 93.60, *p* < 0.05 for *creativity*; *χ*^2^(7) = 82.75, *p* < 0.05 for *curiosity*; *χ*^2^(7) = 45.77, *p* < 0.05 for *fairness*; *χ*^2^(7) = 64.65, *p* < 0.05 for *forgiveness*; *χ*^2^(7) = 284.89, *p* < 0.05 for *gratitude*; *χ*^2^(7) = 91.94, *p* < 0.05 for *honesty*; *χ*^2^(7) = 501.51, *p* < 0.05 for *hope*; *χ*^2^(7) = 82.37, *p* < 0.05 for *humor*; *χ*^2^(7) = 211.03, *p* < 0.05 for *perseverance*; *χ*^2^(7) = 61.70, *p* < 0.05 for *judgment*; *χ*^2^(7) = 54.96, *p* < 0.05 for *kindness*; *χ*^2^(7) = 118.39, *p* < 0.05 for *leadership*; *χ*^2^(7) = 43.38, *p* < 0.05 for *love of learning*; *χ*^2^(7) = 26.44, *p* < 0.05 for *humility*; *χ*^2^(7) = 28.88, *p* < 0.05 for *perspective*; *χ*^2^(7) = 232.17, *p* < 0.05 for *self-regulation*; *χ*^2^(7) = 98.37, *p* < 0.05 for *social intelligence*; *χ*^2^(7) = 87.83, *p* < 0.05 for *spirituality*; *χ*^2^(7) = 408.61, *p* < 0.05 for *zest*; *χ*^2^(7) = 21.20, *p* < 0.05 for *appreciation of beauty and excellence*.

**Dunn Bonferroni Post Hoc Analyses**

***Creativity.*** SDG (mean rank difference [MRD] = -786.13, *p*<.05) and EDG (MRD = -799.96, *p*<.05) had significantly lower creativity scores than IDG. SLDG (MRD = 774.16, *p*<.05), TBIG (MRD = 993.74, *p*<.05), MDG (MRD = 669.05, *p*<.05) had significantly higher creativity scores than IDG. OHIG (MRD = 348.96, *p*<.05), SLDG (MRD = 787.99, *p*<.05), TBIG (MRD = 1007.57, *p*<.05), and MDG (MRD = 682.89, *p*<.05) had significantly higher creativity scores than EDG. MDG had significantly higher creativity scores than OHIG (MRD = 333.93, *p*<.05).

***Curiosity.*** EDG has significantly lower curiosity scores than IDG (MRD = -778.28, *p*<.05) and SDG (MRD = -529.34, *p*<.05). OIG (MRD = 614.35, *p*<.05), OHIG (MRD = 543.80, *p*<.05), SLDG (MRD = 1041.68, *p*<.05), TBIG (MRD = 1104.92, *p*<.05), and MDG (MRD = 576.68, *p*<.05) had significantly higher curiosity scores than EDG. SLDG had significantly higher curiosity scores compared with OHIG (MRD = 497.87, *p*<.05).

***Judgment.*** IDG had significantly higher judgment scores than EDG (MRD = -984.61, *p*<.05) and SLD (MRD = -854.16, *p*<.05). EDG had significantly lower judgment scores than SDG (MRD = -554.23, *p*<.05). OIG (MRD = 652.30, *p*<.05), OHIG (MRD = 536.04, *p*<.05), and MDG (MRD = 362.45, *p*<.05) had significantly higher judgment scores than EDG.

***Love of Learning.*** EDG had significantly lower love of learning scores than IDG (MRD = -760.86, *p*<.05) and SDG (MRD = -383.00, *p*<.05). OHIG (MRD = 434.17, *p*<.05), TBIG (MRD = 1248.05, *p*<.05), and MDG (MRD = 455.04, *p*<.05) had significantly higher love of learning scores than EDG.

***Perspective.*** OID (MRD = 557.12, *p*<.05), OHIG (MRD = 371.08, *p*<.05), and MDG (MRD = 308.98, *p*<.05) had significantly higher perspective scores than EDG.

***Bravery***. TBIG had significantly higher bravery scores than IDG (MRD = 1092.57, *p*<.05) and OHIG (MRD = 923.44, *p*<.05). OHIG (MRD = 368.88, *p*<.05), SLDG (MRD = 695.86, *p*<.05), TBIG (MRD = 1292.33, *p*<.05), and MDG (MRD = 617.02, *p*<.05) had significantly higher bravery scores than SDG. OIG (MRD = 555.94, *p*<.05), OHIG (MRD = 420.38, *p*<.05), SLDG (MRD = 747.36, *p*<.05), TBIG (MRD = 1343.83, *p*<.05), and MDG (MRD = 668.51, *p*<.05) had significantly higher bravery scores than EDG.

***Honesty.*** TBIG had significantly higher honesty scores than IDG (MRD = 1431.01, *p*<.05), EDG (MRD = 1610.94, *p*<.05), OHIG (MRD = 1084.67, *p*<.05), and SLDG (MRD = 1540.90, *p*<.05). EDG (MRD = -786.54, *p*<.05), SLDG (MRD = -716.51, *p*<.05), and MDG (MRD = -389.84, *p*<.05) had significantly lower scores than SDG. OIG (MRD = 811.60, *p*<.05), OHIG (MRD = 526.26, *p*<.05), TBIG (MRD = 1610.94, *p*<.05), and MDG (MRD = 396.69, *p*<.05) had significantly higher honesty scores than EDG. SLDG had significantly lower honesty scores than OIG (MRD = -741.57, *p*<.05). MDG group had significantly lower honestly scores than TBIG (MRD = -1214.24, *p*<.05).

***Perseverance.*** IDG had significantly lower perseverance scores compared with SDG (MRD = 678.69, *p*<.05), OIG (MRD = 808.55, *p*<.05), and TBIG (MRD = 1113.44, *p*<.05). SDG had significantly higher perseverance scores than EDG (MRD = -1206.97, *p*<.05), OHIG (MRD = -859.24, *p*<.05), SLDG (MRD = -1298.04, *p*<.05), and MDG (MRD = -1178.63, *p*<.05). EDG had significantly lower scores than OIG (MRD = 1336.83, *p*<.05), OHIG (MRD = 347.73, *p*<.05), and TBIG (MRD = 1641.72, *p*<.05). OIG had significantly higher perseverance scores than OHIG (MRD = -989.09, *p*<.05), SLDG (MRD = -1427.90, *p*<.05), and MDG (MRD = -1308.49, *p*<.05). TBIG had significantly higher perseverance scores than OHIG (MRD = 1293.98, *p*<.05) and SLDG (MRD = 1732.78, *p*<.05). MDG had significantly lower perseverance scores than OHIG (MRD = -319.39, *p*<.05) and TBIG (MRD = -1613.37, *p*<.05).

***Zest.*** Results revealed that SDG (MRD = 773.65, *p*<.05), SLDG (MRD = 1051.00, *p*<.05), and TBIG (MRD = 1086.37, *p*<.05) had significantly higher zest scores than IDG. SDG had significantly higher zest scores than EDG (MRD = -1718.51, *p*<.05), OHIG (MRD = -706.54, *p*<.05), and MDG (MRD = -1227.31, *p*<.05). EDG group had significantly lower zest scores than IDG (MRD = -944.86, *p*<.05). OIG (MRD = 1649.37, *p*<.05), OHIG (MRD = 1011.96, *p*<.05), SLDG (MRD = 1995.86, *p*<.05), TBIG (MRD = 2031.24, *p*<.05), and MDG (MRD = 491.20, *p*<.05) had significantly higher zest scores than EDG. OIG had significantly higher zest scores than OHIG (MRD = -637.40, *p*<.05) and MDG (MRD = -1158.17, *p*<.05). SLDG (MRD = 983.89, *p*<.05) and TBIG (MRD = 1019.27, *p*<.05) had significantly higher zest scores than OHIG. MDG had significantly lower zest scores than OIG (MRD = -520.76, *p*<.05), SLDG (MRD = -1504.66, *p*<.05), and TBIG (MRD = -1540.04, *p*<.05).

***Kindness.*** MDG had significantly higher kindness scores than IDG (MRD = 824.70, *p*<.05), SDG (MRD = 550.97, *p*<.05), EDG (MRD = 543.10, *p*<.05), and OHIG (MRD = 325.47, *p*<.05).

***Love.*** SDG (MRD = 835.49, *p*<.05), OIG (MRD = 940.90, *p*<.05), OHIG (MRD = 1091.42, *p*<.05), SLDG (MRD = 1055.14, *p*<.05), TBIG (MRD = 1484.34, *p*<.05), and MDG (MRD = 937.68, *p*<.05) had significantly higher loves scores than IDG. OHIG (MRD = 460.74, *p*<.05) and MDG (MRD = 307.00, *p*<.05) had significantly higher love scores than EDG.

***Social Intelligence.*** SDG (MRD = 950.94, *p*<.05), EDG (MRD = 1355.26, *p*<.05), OIG (MRD = 1714.35, *p*<.05), OHIG (MRD = 1580.24, *p*<.05), SLDG (MRD = 1760.71, *p*<.05), TBIG (MRD = 1777.90, *p*<.05), and MDG (MRD = 1395.42, *p*<.05) had significantly higher social intelligence scores than IDG. EDG (MRD = 404.32, *p*<.05), OIG (MRD = 763.41, *p*<.05), OHIG (MRD = 629.30, *p*<.05), SLDG (MRD = 809.77, *p*<.05), and MDG (MRD = 444.48, *p*<.05) had significantly higher social intelligence scores than SDG.

***Fairness.*** SDG (MRD = -406.02, *p*<.05) had significantly higher fairness scores than EDG. OHIG (MRD = 482.72, *p*<.05), SLDG (MRD = 579.88, *p*<.05), TBIG (MRD = 1122.81, *p*<.05), and MDG (MRD = 398.17, *p*<.05) had significantly higher fairness scores than EDG.

***Leadership.*** SDG (MRD = 1014.15, *p*<.05), OIG (MRD = 1597.75, *p*<.05), OHIG (MRD = 1071.16, *p*<.05), SLDG (MRD = 1357.93, *p*<.05), TBIG (MRD = 1755.69, *p*<.05), and MDG (MRD = 741.73, *p*<.05) had significantly higher leadership scores than IDG. EDG (MRD = -547.95, *p*<.05) had significantly lower leadership scores than SDG. OIG (MRD = 1131.55, *p*<.05), OHIG (MRD = 604.96, *p*<.05), SLDG (MRD = 891.74, *p*<.05), and TBIG (MRD = 1289.50, *p*<.05) had significantly higher leadership scores than EDG. MDG had significantly lower leadership scores than OIG (MRD = -856.02, *p*<.05), OHIG (MRD = -329.43, *p*<.05), SLDG (MRD = -616.20, *p*<.05), and TBIG (MRD = -1013.96, *p*<.05).

***Teamwork.*** SDG (MRD = 925.04, *p*<.05), SLDG (MRD = 870.14, *p*<.05), and TBIG (MRD = 1271.59, *p*<.05) had significantly higher teamwork scores than IDG. EDG (MRD = -717.58, *p*<.05), OHIG (MRD = -357.88, *p*<.05), and MDG (MRD = -609.09, *p*<.05) had significantly lower teamwork scores than SDG. OHIG (MRD = 359.70, *p*<.05), SLDG (MRD = 662.689, *p*<.05), and TBIG (MRD = 1064.13, *p*<.05) had significantly higher teamwork scores than EDG. SLDG (MRD = -554.19, *p*<.05) and TBIG (MRD = -955.64, *p*<.05) had significantly lower teamwork scores than MDG.

***Forgiveness.*** EDG (MRD = -559.36, *p*<.05) had significantly lower forgiveness score than SDG. OIG (MRD = 743.90, *p*<.05), OHIG (MRD = 610.89, *p*<.05), SLDG (MRD = 742.67, *p*<.05), and MDG (MRD = 385.33, *p*<.05) had significantly higher forgiveness scores than EDG.

***Humility.*** EDG (MRD = -353.68, *p*<.05), SLDG (MRD = -676.90, *p*<.05), and MDG (MRD = -391.21, *p*<.05) had significantly lower humility scores than SDG.

***Prudence*.** SLDG had significantly lower prudence scores compared with IDG (MRD = -848.20, *p*<.05), SDG (MRD = -1255.60, *p*<.05), EDG (MRD = -566.33, *p*<.05), OIG (MRD = -1120.16, *p*<.05), and OHIG (MRD = -803.79, *p*<.05). EDG (MRD = -689.26, *p*<.05), OHIG (MRD = -451.80, *p*<.05), and MDG (MRD = -723.13, *p*<.05) had significantly lower prudence scores compared to SDG. OIG (MRD = 553.82, *p*<.05) had significantly higher prudence scores compared to EDG. MDG had significantly lower prudence scores compared to OIG (MRD = -587.69, *p*<.05) and OHIG (MRD = -271.33, *p*<.05). TBIG (MRD = 1155.46, *p*<.05) and MDG (MRD = 532.46, *p*<.05) had significantly higher prudence scores compared to SLD.

***Self-Regulation.*** EDG (MRD = -903.97, *p*<.05), SLDG (MRD = -855.74, *p*<.05), and MDG (MRD = -712.01, *p*<.05) had significantly lower self-regulation scores compared with IDG. EDG (MRD = -1330.73, *p*<.05), OHIG (MRD = -844.31, *p*<.05), SLDG (MRD = -1282.49, *p*<.05), and MDG (MRD = -1138.76, *p*<.05) had significantly lower self-regulation scores compared with SDG. OIG (MRD = 1489.82, *p*<.05), OHIG (MRD = 486.42, *p*<.05), and TBIG (MRD = 1708.55, *p*<.05) had significantly higher self-regulation scores compared with EDG. OHIG (MRD = -1003.40, *p*<.05), SLDG (MRD = -1441.59, *p*<.05), and MDG (MRD = -1297.86, *p*<.05) had significantly lower self-regulation scores than OIG. TBIG had significantly higher self-regulation scores compared with OHIG (MRD = 1222.13, *p*<.05) and SLDG (MRD = 1660.31, *p*<.05). SLD had significantly lower self-regulation scores than OHIG (MRD = -294.45, *p*<.05) and TBIG (MRD = -516.76, *p*<.05).

***Appreciation of Beauty and Excellence.*** Only MDL (MRD = 359.16, *p*<.05) had significantly higher appreciation of beauty and excellence scores compared with SDG.

***Gratitude.*** SDG (MRD = 1152.21, *p*<.05), OIG (MRD = 1206.60, *p*<.05), OHIG (MRD = 742.09, *p*<.05), and TBIG (MRD = 1840.50, *p*<.05) had significantly higher gratitude scores compared with IDG. EDG (MRD = -1483.29, *p*<.05), OHIG (MRD = -410.12, *p*<.05), and MDG (MRD = -820.25, *p*<.05) had significantly lower gratitude scores compared with SDG. OIG (MRD = 1537.68, *p*<.05), OHIG (MRD = 1073.17, *p*<.05), SLDG (MRD = 1027.43, *p*<.05), TBIG (MRD = 2171.57, *p*<.05), and MDG (MRD = 663.03, *p*<.05) had significantly higher gratitude scores compared with EDG. MDG had significantly lower gratitude scores compared with OIG (MRD = -874.64, *p*<.05), OHIG (MRD = -410.13, *p*<.05), TBIG (MRD = -1508.54, *p*<.05). TBIG had significantly higher gratitude scores compared with OHIG (MRD = 1098.40, *p*<.05) and SLD (MRD = 1144.13, *p*<.05).

***Hope.*** SDG (MRD = 752.12, *p*<.05), OIG (MRD = 957.87, *p*<.05), and TBIG (MRD = 1147.47, *p*<.05) had significantly higher hope scores compared with IDG. EDG (MRD = -1950.17, *p*<.05), OHIG (MRD = -523.93, *p*<.05), and MDG (MRD = -1190.89, *p*<.05) had significantly lower hope scores compared with SDG. IDG (MRD = 1198.04, *p*<.05), OIG (MRD = 2155.91, *p*<.05), OHIG (MRD = 1426.23, *p*<.05), SLDG (MRD = 1574.00, *p*<.05), TBIG (MRD = 2345.51, *p*<.05), and MDG (MRD = 759.27, *p*<.05) had significantly higher hope scores compared with EDG. OHIG (MRD = -729.68, *p*<.05) had significantly lower hope score compared with OIG. TBIG had significantly higher hope scores compared with OHIG (MRD = 919.28, *p*<.05). MDG had significantly lower hope scores compared with OIG (MRD = -1396.64, *p*<.05), OHIG (MRD = -666.95, *p*<.05), SLDG (MRD = -814.72, *p*<.05), and TBIG (MRD = -1586.23, *p*<.05).

***Humor.*** SDG (MRD = 589.91, *p*<.05), OIG (MRD = 1028.37, *p*<.05), OHIG (MRD = 644.47, *p*<.05), SDLG (MRD = 781.98, *p*<.05), and MDG (MRD = 561.52, *p*<.05) had significantly higher humor scores compared with EDG.

***Spirituality.*** SDG (MRD = 668.85, *p*<.05), OIG (MRD = 851.98, *p*<.05), and TBIG (MRD = 1316.10, *p*<.05) had significantly higher spirituality scores compared with IDG. SDG (MRD = 691.03, *p*<.05), OIG (MRD = 874.15, *p*<.05), OHIG (MRD = 463.18, *p*<.05), TBIG (MRD = 1338.27, *p*<.05), and MDG (MRD = 643.743, *p*<.05) had significantly higher spirituality scores compared with EDG. TBIG had significantly higher spirituality score compared with SLDG (MRD = 1043.52, *p*<.05).
